# Multi‐Environment Genomic Analysis Reveals Limited Concordance Between Sorghum Leaf Blight Incidence and Severity

**DOI:** 10.1002/pei3.70214

**Published:** 2026-11-02

**Authors:** Ezekiel Ahn, Insuck Baek, Louis K. Prom, Moon S. Kim, Lyndel W. Meinhardt, Clint Magill

**Affiliations:** ^1^ Department of Agriculture, Sustainable Perennial Crops Laboratory Agricultural Research Service Beltsville Maryland USA; ^2^ Department of Agriculture, Environmental Microbial and Food Safety Laboratory Agricultural Research Service Beltsville Maryland USA; ^3^ United States Department of Agriculture Insect Control and Cotton Disease Research, Agricultural Research Service, Southern Plains Agricultural Research Center College Station Texas USA; ^4^ Department of Plant Pathology and Microbiology Texas A&M University College Station Texas USA

**Keywords:** ACAT, disease incidence, disease severity, *Exserohilum turcicum*, GEMMA, genome‐wide association study, leaf blight, multi‐environment analysis, *Sorghum bicolor*

## Abstract

Leaf blight caused by *Exserohilum turcicum* constrains sorghum production in semi‐arid environments. Disease incidence and severity are related, but their relationship need not imply shared resistance loci. Records from five locations in Niger and Senegal comprised 1339 paired plot observations. Field‐stratum‐adjusted accession values were estimated before rank‐based inverse‐normal transformation. The primary population contained 100 accessions evaluated in at least four locations; all 102 genotype‐matched accessions were included in a sensitivity analysis. Genome‐wide efficient mixed‐model association (GEMMA) score tests used leave‐one‐chromosome‐out kinship matrices, and trait‐level *p*‐values were combined with the aggregated Cauchy association test (ACAT) and the Simes procedure. Adjusted incidence and standard severity were moderately correlated (Pearson *r* = 0.429); the correlation was lower after conditioning severity on incidence (*r* = 0.066). Across 567,758 single‐nucleotide polymorphisms, ACAT had a genomic inflation factor of 1.014 and a minimum *p*‐value of 2.64 × 10^−5^. No marker passed the Benjamini–Hochberg 10% false‐discovery‐rate criterion for either trait or either combination; likelihood‐ratio analyses also produced no discoveries at this threshold. Incidence–severity association‐profile concordance was weak (Pearson *r* = 0.127; Spearman *ρ* = 0.094). Top‐ranked overlap was 5.65‐fold at 1% and 2.24‐fold at 5% relative to the marker‐count expectation, a descriptive comparison that does not account for linkage disequilibrium. ACAT and Simes rankings were similar (*ρ* = 0.895). Ten top‐ranked, genotype‐equivalence‐aware, linkage‐disequilibrium‐clumped regions are presented as exploratory candidates. Phenotypic association was therefore stronger than detectable sharing of marker‐association evidence. Limited sample size and sensitivity to phenotype definition, field composition and test choice constrain locus‐level conclusions.

## 1 Introduction

Sorghum ( *Sorghum bicolor* (L.) Moench) supports food and feed production across drought‐prone regions because of its C_4_ physiology and adaptation to heat and water limitation (Mundia et al. 2019; Hao et al. 2021; George et al. 2022; Hossain et al. 2022). Foliar disease can erode that resilience when susceptible material is grown under environments favorable for infection and lesion expansion. Leaf blight caused by *Exserohilum turcicum* is influenced by host genotype, pathogen pressure and local weather, making resistance difficult to measure consistently across field sites (Zhang et al. 2020; Khaskheli et al. 2025).

West African evaluations have provided a multi‐location resource for examining sorghum leaf blight resistance. An analysis of Nigerien and Senegalese germplasm evaluated at three Senegalese sites reported incidence‐associated markers (Prom et al. 2023). A later analysis of the panel at two Nigerien sites combined genome‐wide association and machine‐learning rankings for incidence and severity (Prom et al. 2026). The two reports used related germplasm but distinct field evaluations, and incidence and severity were analyzed largely as separate outcomes.

Incidence measures the proportion of diseased plants, whereas severity describes the extent of symptoms on the assessed plant material. These measurements can covary for biological reasons and because of their scoring definitions. Field imbalance, population structure, repeated observations, and small sample size add uncertainty to association mapping (Korte and Farlow 2013; Manchia et al. 2013; Larkan et al. 2016; Demirjian et al. 2023). Multi‐trait approaches offer ways to combine related phenotypes (Thoen et al. 2017; Ahn et al. 2023), but a combined *p*‐value does not establish pleiotropy or shared causal effects (Chebib and Guillaume 2021).

Plot‐level records from the five locations were reanalyzed to examine phenotypic dependence alongside marker‐association concordance. Accession values were estimated across field strata before transformation, and severity was analyzed with and without adjustment for incidence and with restriction to disease‐positive plots. Kinship‐adjusted association profiles were combined with the aggregated Cauchy association test (ACAT) and compared with the Simes procedure. Calibration, detectable‐effect limits, genotype‐equivalent markers, and leave‐one‐location‐out sensitivity were evaluated to distinguish reproducible statistical patterns from unsupported locus‐level claims.

## 2 Materials and Methods

### 2.1 Field Records and Phenotype Definitions

Leaf blight records were obtained from the 2022 field evaluations reported for Senegal (Prom et al. 2023) and Niger (Prom et al. 2026). The sites were Maradi and Bengou in Niger and Kolda, Ndiaganiao, and Kaymor in Senegal. The source evaluations used replicated field plots. Of 1520 raw rows, 1339 had complete incidence–severity pairs, representing 104 accession identifiers. Location, country, year, replicate, block, plot, and plant‐count information were retained where recorded. Blank cells and missing‐value tokens were distinguished from numeric zero. Disease values were reconciled with the consolidated leaf‐blight input; one raw severity entry of 145.5 was represented there as 15.0. That documented input value was retained without inferring a replacement midpoint.

Incidence was expressed as the percentage of assessed plants showing leaf blight within each row. Severity scoring included only plants exhibiting leaf blight symptoms. The compiled dataset contained one overall severity value per plot, expressed on the percentage‐midpoint scale derived from the published 0–11 ratings (Prom et al. 2023, 2026). Individual plant‐ and leaf‐level component scores were not available for reanalysis. Among the 1339 complete pairs, all 56 plots with zero incidence also had zero severity, and neither trait was zero when the other was positive. Two sensitivity analyses examined this dependence: adjustment of severity for plot‐level incidence and restriction to plots with incidence > 0. The disease‐positive‐only analysis excluded plots without symptomatic plants; it did not change which plants contributed to the original severity assessment.

### 2.2 Multi‐Environment Phenotype Reconstruction

Accession values were estimated on the untransformed scale using accession and field stratum as fixed effects. Field stratum was defined by the observed location–replicate–block combination, yielding 33 strata. Equal‐weighted marginal accession means were calculated over these strata. For plot *k* of accession *i* in stratum *j*, the model was as follows:

$$y_{\mathrm{ijk}}=\mu +G_{i}+S_{j}+\varepsilon_{\mathrm{ijk}}.$$

Here, *y*_*ijk*_ is the plot value, *μ* is the intercept, *G*_*i*_ is the accession effect, *S*_*j*_ is the field‐stratum effect and *ε*_*ijk*_ is the residual. Incidence‐adjusted severity added centred plot‐level incidence as a covariate; accession means were evaluated at the mean incidence. Disease‐positive‐only severity used the same stratum model after restricting observations to incidence > 0. A rank‐based inverse‐normal transformation (INT) was applied to accession estimates, not to individual plot records (Beasley et al. 2009). Average ranks were used for ties, with transformed value Φ^−1^[(*R*_*i*_ − 0.5)/*N*], where *R*_*i*_ is the rank, *N* is the number of finite accession estimates and Φ^−1^ is the standard‐normal quantile function. Ranks were calculated over the 104 finite accession estimates for each reported trait before restriction to the genotype‐matched analysis populations. The accession‐by‐environment interaction and unequal uncertainty of accession estimates were not modeled in the subsequent marker scans.

Of the 104 accession identifiers, 102 matched the genotype resource. The primary set contained 100 accessions represented in at least four locations, limiting reliance on sparse field records. The two matched accessions represented at one location were retained in an *n* = 102 sensitivity analysis. Accession coverage and inclusion are listed in Data S1 (Population_Definition).

### 2.3 Genotype Filtering and Genomic Structure

Genotypes were obtained from the public sorghum single‐nucleotide polymorphism (SNP) resource associated with Prom et al. (2026). Biallelic markers on chromosomes 1–10 were filtered in the 102 genotype‐matched accessions at minor‐allele frequency (MAF) ≥ 0.05 and missing‐call rate ≤ 0.20, following standard marker‐quality principles (Anderson et al. 2010). Missing dosages were replaced by the marker mean. A common set of 567,758 markers was retained for the primary and sample‐inclusion sensitivity analyses. Reported MAF values refer to this quality‐control population.

The genomic‐structure set contained 8566 markers. Markers were first thinned to one candidate per 20‐kb bin and then pruned within 250‐kb windows at squared dosage correlation *r* ^2^ ≥ 0.20. Centred dosages were scaled by the square root of 2*f* (1 − *f*), where *f* is the allele frequency, before constructing genomic relationship matrices. A separate leave‐one‐chromosome‐out (LOCO) matrix excluded structure markers on the chromosome under test. Principal components (PCs) from the global relationship matrix were used in sensitivity analyses (Price et al. 2006). The same marker set and primary‐population PCs were used for the ordinary least‐squares (OLS) transformation diagnostics (Data S1, Structure_Markers and Structure_PCs).

### 2.4 Single‐Trait Mixed‐Model Association Tests

Association testing used genome‐wide efficient mixed‐model association (GEMMA), version 0.98.5 (Zhou and Stephens 2012). For a marker on chromosome *c*, the model was as follows:

$$y=\mathrm{X\alpha}+\mathrm{g\beta}+u+\varepsilon ,u∼N\left(0,{\sigma_{g}}^{2}K_{-c}\right),\varepsilon ∼N\left(0,{\sigma_{e}}^{2}I\right).$$

The response *y* contains transformed accession estimates; *X* contains the intercept; *g* is additive marker dosage; *β* is the marker effect; *K*_−c_ is the LOCO genomic relationship matrix; and *I* is the identity matrix. The variance components *σ*_g_^2^ and *σ*_e_^2^ describe the random genomic and residual terms. Stored dosages *d* = 0, 1 and 2 were exported as synthetic A/A, A/G and G/G genotypes. GEMMA coefficients refer to one additional copy of synthetic allele1 A (*g* = 2 − *d*), in inverse‐normal phenotype units. These encoding labels do not identify the original SNP nucleotides or favorable alleles. Score tests, which evaluate association under the fitted null covariance model, were used for primary summaries. Likelihood‐ratio tests (LRTs) provided a sensitivity comparison, and Wald results were retained to show test‐dependent behavior. This reporting hierarchy followed the comparison of the three tests; it was not chosen before inspection of the data. Disagreement between tests was not treated as proof that one test was correctly calibrated. Effect estimates and standard errors (SEs) from the alternative‐model restricted maximum likelihood (REML) fit in GEMMA mode 4 were reported separately from score‐test *p*‐values. Descriptive 95% confidence intervals (CIs) were calculated as *β* ±1.96 SE; they are unadjusted Wald intervals, not inversions of the score test or simultaneous intervals.

### 2.5 Dual‐Trait *p*‐Value Combination and Concordance

Incidence and severity score‐test *p*‐values were combined at each marker with the aggregated Cauchy association test (ACAT) using equal weights (Liu et al. 2019; Liu and Xie 2020). ACAT combines dependent *p*‐values without requiring estimation of their correlation and can retain evidence dominated by one small component *p*‐value. The Simes procedure supplied an alternative combination (Simes 1986): for two ordered *p*‐values, *p*_Simes_ = min [1, 2*p*_(1)_, *p*_(2)_]. Neither approach requires both traits to be associated. Consequently, combined evidence and cross‐trait concordance were described separately, without interpreting either as a direct estimate of genetic correlation or proof of pleiotropy.

Concordance was summarized by Pearson and Spearman correlations between −log_10_(*p*) profiles. At the top 1%, 5%, and 10% of each trait, observed overlap was divided by *k*_INC_ *k*_SEV_/*M*, where the 2 *k* values are the selected marker counts and *M* is the total tested marker count. Jaccard similarity and odds ratios were also tabulated. Marker‐level Fisher exact probabilities were retained in Data S1 for completeness, but linked markers do not constitute independent observations. These overlap statistics were therefore interpreted descriptively rather than as linkage‐disequilibrium‐adjusted evidence against a genome‐wide null.

### 2.6 Multiple Testing and Genotype‐Equivalence Analysis

Benjamini–Hochberg adjusted *p*‐values, denoted *q*, were calculated separately across the 567,758 marker rows for each trait, combination and test, with *q* < 0.10 defining a discovery (Benjamini and Hochberg 1995). The genomic inflation factor *λ*_GC_ was the median of the one‐degree‐of‐freedom *χ* ^2^ equivalents of the *p*‐values divided by the corresponding null median (Devlin and Roeder 1999). It was used as a distributional diagnostic, not as a correction to the *p*‐values or a guarantee of tail calibration. Exact and allele‐complement dosage patterns were grouped within chromosomes to identify duplicated LOCO tests; a parallel grouping across chromosomes described marker aliases. False‐discovery‐rate (FDR) calculations on one representative per chromosome‐specific class provided a multiplicity sensitivity analysis. Global aliases were not interpreted as independent biological replication.

### 2.7 Exploratory Candidate Regions and Nearby Genes

Exploratory candidates were selected by grouping exact and allele‐complement genotype aliases before linkage‐disequilibrium (LD) clumping. Index markers required *p* ≤ 1 × 10^−4^ in score‐based ACAT or severity, secondary markers required *p* ≤ 1 × 10^−3^, and clumping used a ±1‐Mb window with *r* ^2^ ≥ 0.20 in the primary 100‐accession genotypes. The top 10 entries from the combined candidate list were retained for display; 10 is a reporting limit, not a discovery count. These local clumps do not establish complete statistical independence across the genome. Regional profiles were plotted within ±1 Mb (Pruim et al. 2010). Gene features from the *Sorghum bicolor* v3.1.1 annotation were listed within 50 kb of an index marker; the nearest gene was reported separately when that interval contained none. Proximity was not treated as functional validation.

### 2.8 Sensitivity and Detectable‐Effect Analyses

Sensitivity analyses included three genomic PCs, all 102 matched accessions, incidence‐adjusted severity, disease‐positive‐only severity and omission of each location in turn. ACAT and Simes were recalculated within these comparisons. Location‐omission support was summarized through score‐test *p*‐values and rank concordance. For the 10 displayed candidates, all‐location coefficients were additionally obtained with GEMMA mode 3 and compared with the mode‐3 coefficients after each location omission. Both sides used covariance ratios estimated under the null model by maximum likelihood and the same synthetic allele coding. Effect‐direction agreement was tabulated separately from the alternative‐model REML effects and Wald CIs (Data S1, LOO_Effects and LOO_Effect_Summary). To examine transformation and population correction separately, diagnostic ordinary least‐squares (OLS) scans compared adjusted raw and INT phenotypes using 0, 1, 2, 3 or 5 PCs in the same 100‐accession population. Both phenotype and marker dosage were projected against the intercept and selected PCs. Missing dosages were mean‐imputed in the 102‐accession genotype quality‐control set before subsetting, and two‐sided *t‐*tests used *n* – rank (*X*) − 1 residual degrees of freedom. The PC basis used the same 8566 variance‐standardized, LD‐pruned structure markers as the primary project. Reconstructing the PCs from the specified genotype rows reproduced their nested covariate spaces before the OLS scans. These scans were not used to select reported loci (Data S1, Transformation_Checks). Detectable per‐allele effects, expressed in residual standard deviations, were calculated using a two‐sided noncentral‐*t* approximation for *n* = 100 and 102, 80% and 90% power, and allele frequencies *f* = 0.05–0.50. The approximation used *n* − 2 residual degrees of freedom and noncentrality *β*_res_√[*n* × 2*f*(1 − *f*)], where *β*_res_ = *β*/*σ*_e_. Thresholds were 0.05/567,758, 0.05/455,783 and a descriptive *p* = 1 × 10^−5^. This illustrative calculation assumes independent observations and genotype variance 2*f* (1 − *f*); it does not account for kinship, departures from that genotype‐variance assumption in the inbred panel, or uncertainty in the field‐adjusted estimates. The effect unit is not total‐phenotype standard deviation, and the calculation does not estimate empirical mixed‐model power or observed variance explained.

### 2.9 Software and Reproducibility

Data processing used Python 3.11 with NumPy (Harris et al. 2020), SciPy (Virtanen et al. 2020), pandas (McKinney 2010), and statsmodels (Seabold and Perktold 2010). Figures were prepared with Matplotlib (Hunter 2007). Random operations used seed 4,261,991. Input checksums, sample order, phenotype dimensions, and genotype dimensions were recorded. The analysis workflow, including the OLS transformation diagnostics, genotype‐based PC reconstruction, matched‐estimator candidate‐effect comparisons, and residual‐scale detectable‐effect calculations, is archived in Zenodo as version 2.0.2 (DOI: 10.5281/zenodo.22446088). The OLS table uses the primary‐project PC basis, and all‐location candidate coefficients use the same GEMMA mode 3 estimator as the location‐omission coefficients.

## 3 Results

### 3.1 Phenotypic Association Depends on the Severity Definition

The canonical dataset contained 1339 complete plot‐level records: 281 from Maradi, 267 from Bengou, 292 from Kolda, 285 from Ndiaganiao, and 214 from Kaymor (Figure 1a; Figure S1). Of 104 phenotyped accessions, 102 matched the genotype resource and 100 met the primary requirement of representation in at least four locations. Genomic principal components for the primary population are shown in Figure S2.

**FIGURE 1:**
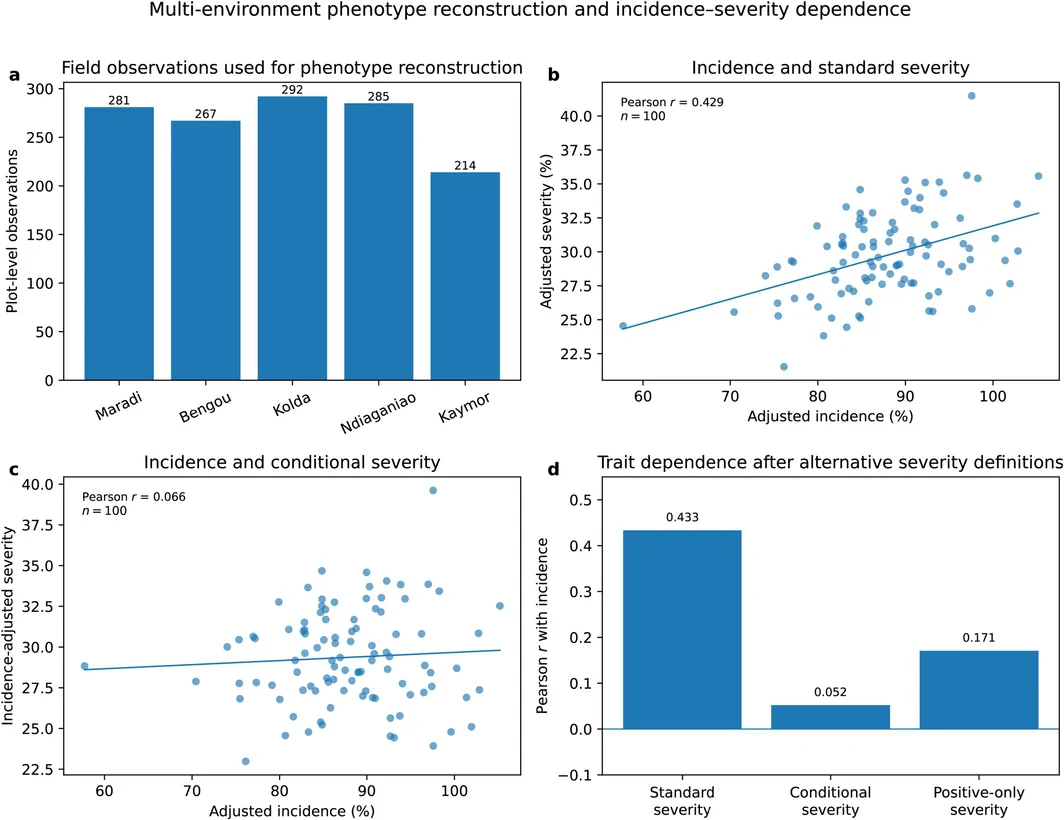
Multi‐environment phenotype summaries and incidence–severity association. (a) Numbers of complete plot records by location; the five counts sum to 1339. (b) Adjusted incidence and standard severity in the 100‐accession primary population. (c) Adjusted incidence and incidence‐adjusted severity in the same population. Each point in (b, c) represents an accession; solid lines are descriptive linear fits, not causal relationships. Insets in (b, c) report Pearson's *r* and the number of accessions (*n*). (d) Pearson correlations between INT incidence and the three INT severity definitions. Adjusted raw values are model estimates rather than individual observed percentages.

Field‐stratum‐adjusted incidence and standard severity were moderately correlated on the raw scale (Pearson *r* = 0.429; Figure 1b). The correlation was lower after including incidence in the severity model (*r* = 0.066; Figure 1c). After INT, correlations with incidence were 0.433 for standard severity, 0.052 for incidence‐adjusted severity, and 0.171 for disease‐positive‐only severity (Figure 1d). These contrasts show how the phenotype summaries change under the three definitions. The low correlation after covariate adjustment does not establish a biologically independent lesion‐expansion trait.

### 3.2 Score and Likelihood‐Ratio Tests Identify no FDR‐Significant Markers

Primary GEMMA LOCO score‐test *λ*_GC_ values were 1.134 for incidence, 0.967 for severity, 1.014 for ACAT and 0.955 for Simes (Table 1; Figure 2; Figure S3). These medians indicate modest inflation for incidence and near‐null medians for the other profiles; they do not rule out residual confounding. Minimum *p*‐values were 1.71 × 10^−5^, 1.32 × 10^−5^ and 2.64 × 10^−5^ for incidence, severity and ACAT, respectively. No marker passed *q* < 0.10 in either single‐trait score test or either combination.

**TABLE 1:** Association‐test summaries for 567,758 SNPs in the 100‐accession primary population. *λ*_GC_ is the median‐based genomic inflation factor. Minimum *p* and minimum *q* are reported separately; *q* is the Benjamini–Hochberg adjusted *p*‐value. No score or likelihood‐ratio analysis produced *q* < 0.10 markers.

Test	Trait/combination	*λ*_GC_	Minimum *p*	Minimum *q*	*q* < 0.10
Score	Incidence	1.134	1.71 × 10^−5^	0.753	0
Score	Severity	0.967	1.32 × 10^−5^	0.988	0
Score	ACAT	1.014	2.64 × 10^−5^	0.889	0
Score	Simes	0.955	2.64 × 10^−5^	0.911	0
LRT	Incidence	1.189	1.33 × 10^−6^	0.154	0
LRT	Severity	1.023	1.16 × 10^−6^	0.283	0
LRT	ACAT	1.101	2.31 × 10^−6^	0.235	0
LRT	Simes	1.054	2.31 × 10^−6^	0.237	0

**FIGURE 2:**
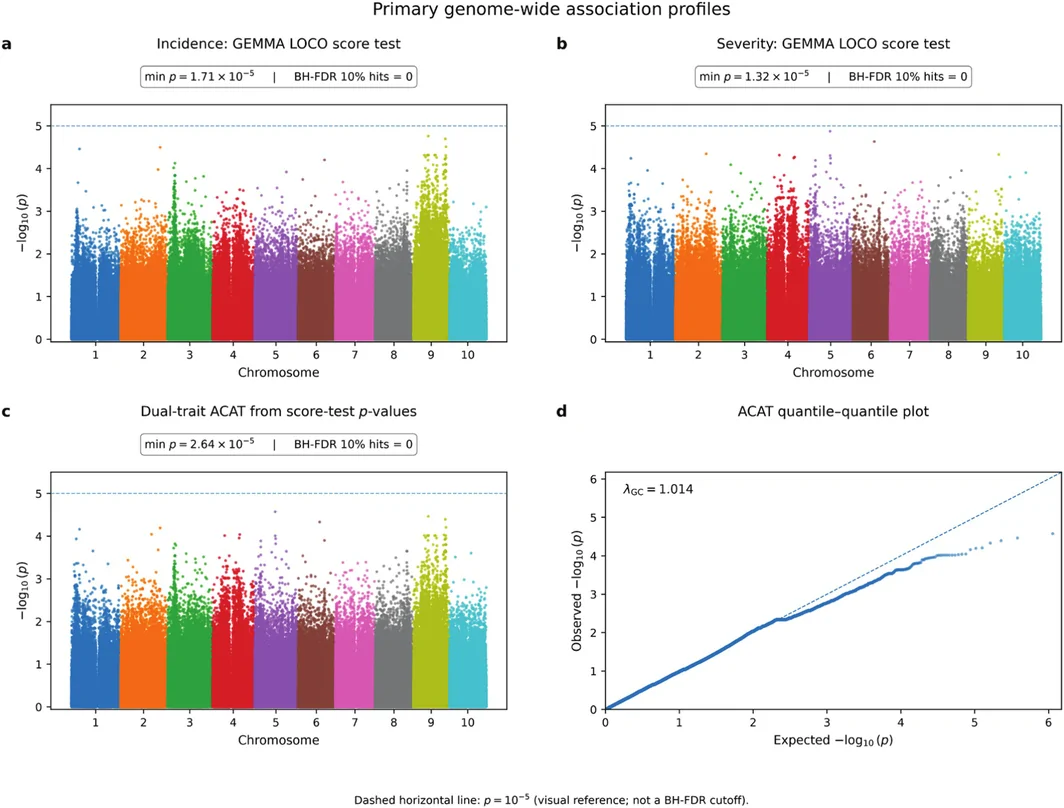
Genome‐wide score‐test profiles. Manhattan plots show −log_10_(*p*) for (a) incidence, (b) severity, and (c) the aggregated Cauchy association test (ACAT). Alternating point colors distinguish chromosomes. The dashed horizontal line at *p* = 1 × 10^−5^ is a plotting reference, not an FDR threshold. Annotations above panels (a–c) report the minimum *p*‐value and the number of markers with *q* < 0.10. (d) ACAT quantile–quantile plot; the diagonal denotes the uniform‐null expectation, and the inset reports *λ*_GC_. All 567,758 SNPs contributed to the summaries. Neither single‐trait score scan nor ACAT or Simes produced FDR discoveries.

LRTs produced smaller minimum *p*‐values but no FDR discoveries: minimum *q* was 0.154 for incidence, 0.283 for severity, 0.235 for ACAT and 0.237 for Simes. Score‐based conditional and positive‐only severity also gave no discoveries, although their *λ*_GC_ values of 0.815 and 0.861 indicated deflation. Wald scans differed substantially: 3906 incidence markers, 3674 severity markers and 7557 ACAT markers passed *q* < 0.10. Those counts are reported as test‐sensitive outcomes, not as established resistance loci (Figure S4; Data S1, Test_Sensitivity). In the raw‐versus‐INT OLS diagnostic, severity *λ*_GC_ was 0.802 versus 0.878 with three PCs and 0.969 versus 1.046 with five PCs; neither setting produced FDR‐significant severity markers. Without PCs, both treatments were strongly inflated. Thus, the observed distribution depended on population correction as well as phenotype treatment (Transformation_Checks).

### 3.3 Association Profiles Show Weak Concordance and Enriched Top‐Ranked Overlap

Incidence and severity score‐test profiles were weakly correlated across the 567,758 markers (Pearson *r* = 0.127; Spearman *ρ* = 0.094; Figure 3a,b). The top 1% sets contained 321 shared markers, giving 5.65‐fold enrichment relative to the marker‐count expectation; the corresponding ratios were 2.24 at 5% and 1.59 at 10% (Figure 3c). These are descriptive rank‐overlap statistics. The correlations are not estimates of genetic correlation, and the enrichment ratios do not incorporate an LD‐preserving null model.

**FIGURE 3:**
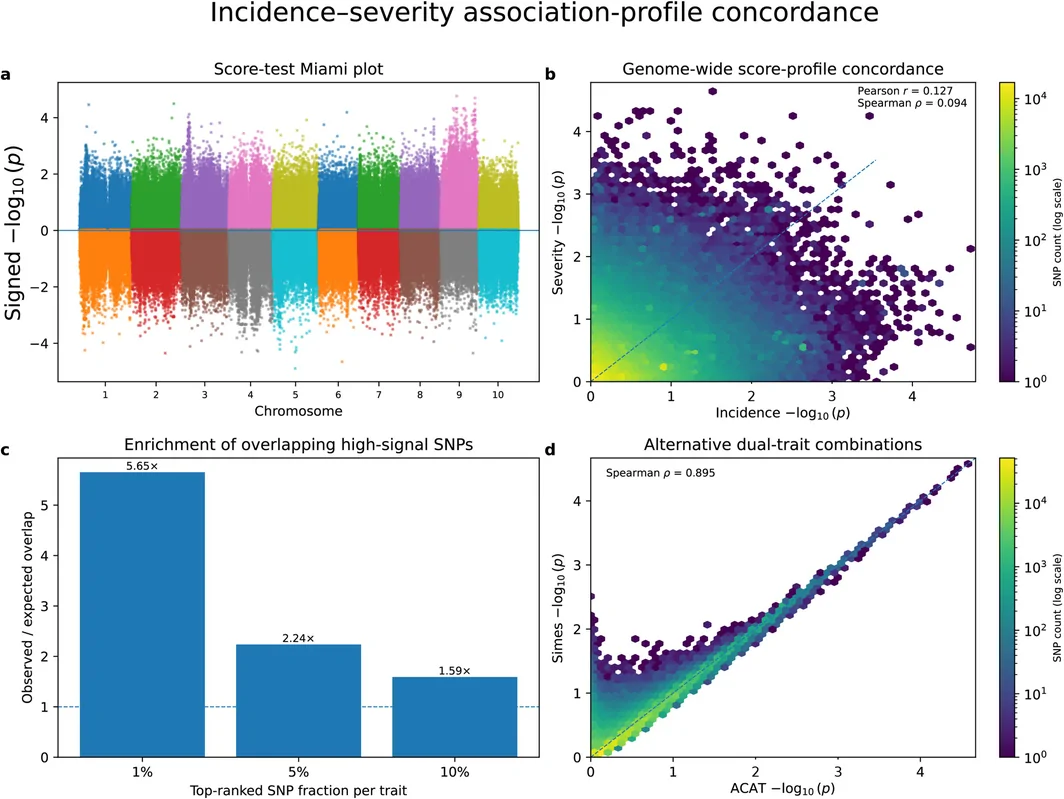
Concordance of incidence and severity score‐test profiles. (a) Miami plot with incidence above zero and severity below zero; vertical values are signed −log_10_(*p*). (b) Hexbin plot of incidence and severity association strengths; color gives marker count on a logarithmic scale. Pearson *r* = 0.127 and Spearman *ρ* = 0.094 describe the marker‐level profiles, not genetic correlation. (c) Observed overlap at the top 1%, 5%, and 10% divided by the marker‐count expectation; the horizontal dashed line denotes a ratio of one. This descriptive reference does not account for LD. (d) ACAT versus Simes association strengths. The inset in (d) reports the Spearman correlation between the ACAT and Simes profiles. Dashed diagonal lines in (b, d) mark equal association strengths. Hexbin color in (d) has the same meaning as in (b). Reported rank statistics use the full marker set.

ACAT and Simes rankings were similar (Spearman *ρ* = 0.895), with top‐1%, top‐5%, and top‐10% Jaccard similarities of 0.954, 0.875, and 0.848 (Figure 3d). Both gave zero FDR discoveries. Adding three PCs changed the ACAT profile more than including all 102 matched accessions: rank correlations with the primary profile were 0.591 and 0.820, respectively. Both comparisons retained the absence of score‐test discoveries (Figure S5; Model_Sensitivity).

### 3.4 Exploratory Regions Show Trait‐Specific and Environment‐Sensitive Support

Grouping exact and allele‐complement genotypes yielded 451,951 global alias classes and 455,783 chromosome‐specific LOCO test classes from the 567,758 marker rows. No chromosome‐specific class passed the score‐ACAT *q* < 0.10 criterion. Alias‐aware clumping at the exploratory thresholds produced 11 ACAT and 10 severity candidate signals; the combined top 10 entries are displayed in Figure 4 and Data S1. Five are summarized in Table 2. S05_34711017 ranked mainly through severity (*p*_SEV_ = 1.32 × 10^−5^; *p*_INC_ = 0.108), whereas S09_24644668 and S02_66258735 ranked mainly through incidence. S06_35920649 retained nominal severity evidence under both alternative severity definitions. Every displayed candidate had ACAT *q* > 0.88.

**FIGURE 4:**
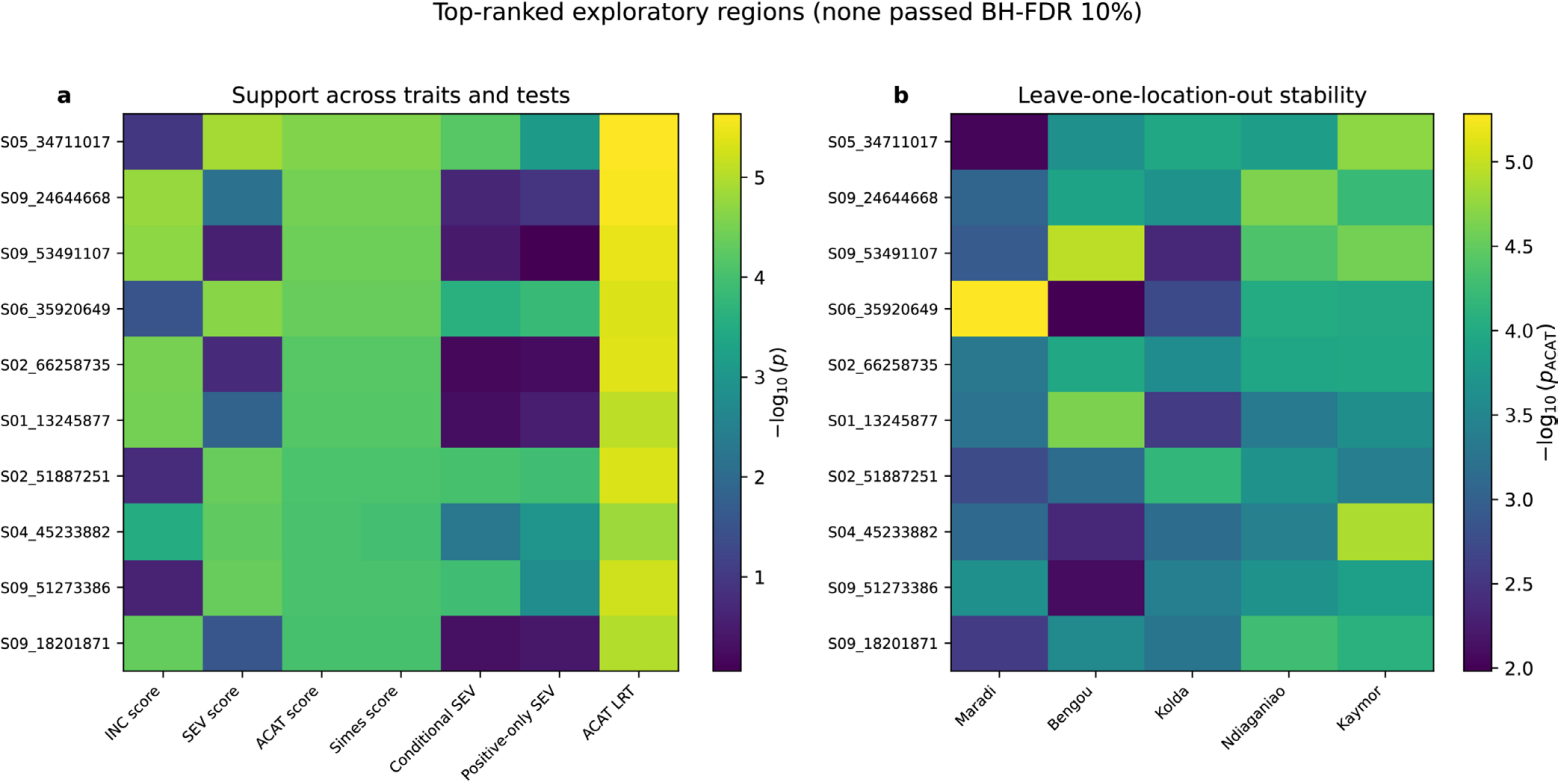
Association‐strength summaries for 10 exploratory candidate entries. Colors represent −log_10_(*p*), with separate color scales for the two panels; color intensity is not an FDR decision. (a) Incidence, standard severity, ACAT, Simes, incidence‐adjusted severity and positive‐only severity use score tests; the final column shows LRT‐based ACAT. (b) Score‐based ACAT after omitting Maradi, Bengou, Kolda, Ndiaganiao, or Kaymor. The same 10 entries are shown in both panels. All had primary score‐based ACAT *q* > 0.88, and none is a confirmed quantitative trait locus.

**TABLE 2:** Five top‐ranked exploratory entries from genotype‐equivalence‐aware LD clumping. All *p*‐values below are official GEMMA LOCO score‐test values. MAF refers to the 102‐accession genotype quality‐control set. No entry satisfies the ACAT FDR criterion. Conditional and positive‐only severity statistics, alternative‐model effects and CIs, aliases and nearby genes for all 10 displayed entries are provided in Data S1.

Index marker	Chr:position (bp)	MAF	*p*_INC_	*p*_SEV_	*p*_ACAT_
S05_34711017	5:34,711,017	0.088	1.08 × 10^−1^	1.32 × 10^−5^	2.64 × 10^−5^
S09_24644668	9:24,644,668	0.176	1.71 × 10^−5^	7.15 × 10^−3^	3.41 × 10^−5^
S09_53491107	9:53,491,107	0.196	2.01 × 10^−5^	2.80 × 10^−1^	4.02 × 10^−5^
S06_35920649	6:35,920,649	0.103	3.09 × 10^−2^	2.31 × 10^−5^	4.61 × 10^−5^
S02_66258735	2:66,258,735	0.299	3.17 × 10^−5^	1.85 × 10^−1^	6.35 × 10^−5^

Leave‐one‐location‐out analyses produced no *q* < 0.10 associations. Genome‐wide ACAT rank correlations with the full analysis ranged from 0.481 after omitting Bengou to 0.798 after omitting Kaymor (Figure S6). Across 50 candidate‐by‐location omissions, ACAT *p*‐values ranged from 5.19 × 10^−6^ to 0.0104 (Figure 4b). Using GEMMA mode 3 on both sides, effect direction agreed in 49 of 50 incidence comparisons and all 50 severity comparisons (LOO_Effects; LOO_Effect_Summary). The only sign change involved incidence at S02_51887251 after omitting Bengou: the coefficient changed from 0.221 to −0.055, with score‐test *p*‐values of 0.171 and 0.738, respectively. These overlapping datasets describe sensitivity to field composition, not independent replication. Regional profiles for four candidates are shown in Figure S7. Nearby‐gene summaries are descriptive and do not establish causal genes.

### 3.5 Sample Size and Environmental Sensitivity Limit Detectable Effects

Under the illustrative noncentral‐*t* approximation, the per‐allele effect required for 80% power at 0.05/567,758 ranged from 2.17 residual standard deviations at *f* = 0.05 to 0.94 at *f* = 0.50 in *n* = 100 (Figure S8; Power). These are effects relative to the assumed residual standard deviation, not effects scaled by total phenotypic variation or observed proportions of variance explained. The large requirements contextualize the absence of FDR discoveries but do not measure the power of the fitted LOCO model. Limited sample size, correlated genotypes, and uncertainty in accession estimates preclude interpreting a non‐significant result as evidence against genetic control.

## 4 Discussion

### 4.1 Phenotypic Dependence Does Not Imply Strong Detectable Genomic Sharing

Incidence and standard severity were moderately correlated after field adjustment, but their score‐test profiles showed only weak concordance. Severity was assessed among symptomatic plants, whereas incidence used all assessed plants as its denominator; the two traits therefore describe different but related aspects of field disease expression. Conditioning severity on incidence reduced the phenotype correlation, as expected from the adjustment itself. Restricting severity to disease‐positive plots also changed that relationship. These results support treating incidence and severity as related measurements with definition‐dependent associations, rather than as interchangeable traits or demonstrably separate causal stages. Enrichment among their strongest marker ranks provides a descriptive lead for follow‐up, not proof of shared causal loci.

Different contributions to symptom occurrence and burden remain biologically plausible (Thoen et al. 2017; Zhang et al. 2020). Incidence and plot‐level severity nevertheless describe related aspects of field disease expression rather than experimentally isolated stages of infection. Incidence adjustment and restriction to disease‐positive plots assess sensitivity to symptom occurrence; neither analysis identifies an independent post‐infection resistance mechanism. ACAT can combine evidence dominated by one trait and cannot establish pleiotropy (Liu and Xie 2020; Chebib and Guillaume 2021). Its close agreement with Simes shows that the main ranking pattern was not specific to one combination rule. Neither approach resolved an FDR‐significant shared locus in the available panel.

### 4.2 Phenotype Reconstruction and Test Calibration Materially Affect Inference

Calibration depended on the phenotype summary, population correction, and association test. The raw‐versus‐INT OLS comparisons showed that deflation could persist without INT and change when the number of PCs changed. In the primary kinship‐adjusted score profiles, ACAT had *λ*_GC_ = 1.014, but a near‐null median alone does not establish correct tail behavior. Wald results were much more significant than score and LRT results. This disagreement is compatible with sensitivity to alternative‐model variance estimation in a small panel, but comparison alone does not establish its cause or identify which test has the correct false‐positive rate. Score‐based primary reporting and LRT sensitivity limit claims to findings not dependent on the extreme Wald tail; they do not replace a dedicated null‐calibration study.

Dense markers also contained many exact or allele‐complement dosage patterns in the 100 accessions. Grouping these patterns separated repeated tests from marker positions and reduced apparent duplication across distant regions. A multiplicity calculation on chromosome‐specific unique tests still gave no score‐ACAT discoveries. Genotype aliases and local LD clumps should nevertheless not be equated with independently replicated biological loci.

### 4.3 Environmental Heterogeneity and Statistical Power Constrain Locus Discovery

Association rankings changed after omitting individual locations, especially Bengou or Maradi. Multi‐environment resistance mapping can be sensitive to field composition (Larkan et al. 2016; McLeod et al. 2023), but the available records do not separate weather, inoculum, and developmental effects. Although matched‐estimator effect directions were preserved in 49/50 incidence and 50/50 severity comparisons, the location‐omission datasets overlap substantially and do not provide independent replication. The varying *p*‐values and lack of FDR discoveries across omissions argue against calling these regions robust cross‐environment quantitative trait loci.

The detectable‐effect calculation further limits interpretation. Even under an independent‐observation approximation, genome‐wide detection at *n* = 100 required large effects relative to the residual standard deviation. Its genotype‐variance assumption may not hold in an inbred diversity panel, and it ignores kinship and field‐estimation error. It should therefore be read as an illustration of sample‐size constraints, not a precise estimate of explained variance or mixed‐model power. INT cannot create the information supplied by additional independent accessions (Korte and Farlow 2013; Demirjian et al. 2023). Larger panels and independent field evaluations are needed to test moderate effects.

### 4.4 Exploratory Regions Provide Hypotheses Rather Than Confirmed Resistance Loci

The exploratory entries summarize trait‐specific rankings: S05_34711017 and S06_35920649 rank mainly through severity, whereas S09_24644668, S09_53491107, and S02_66258735 rank mainly through incidence. S04_45233882 shows nominal evidence from both traits. None meets the FDR criterion. The accompanying effects and CIs are alternative‐model descriptive estimates; their intervals should not be read as score‐test decisions, and selection of top‐ranked markers can exaggerate apparent effects.

The earlier reports analyzed three Senegalese sites or two Nigerien sites and reported markers under different phenotype summaries and statistical models (Prom et al. 2023, 2026). Combining all five sites with field‐stratum adjustment therefore does not constitute a direct replication of either marker list. The added information lies in explicit phenotype construction, incidence‐conditioned severity, test and field‐composition comparisons, and separation of rank‐based candidates from FDR discoveries. The additive stratum model does not estimate accession‐by‐location interactions, and the second‐stage scans do not propagate the full covariance of accession estimates. These limitations affect the precision and interpretation of locus‐level evidence. Nearby‐gene lists identify genomic context only; they do not establish a resistance mechanism or justify immediate marker‐assisted selection.

## 5 Conclusions

Leaf blight incidence and severity were phenotypically associated, but their marker‐association profiles were only weakly concordant in the 100‐accession field panel. Score tests, LRTs, and both *p*‐value combination procedures produced no *q* < 0.10 discoveries. Alternative severity definitions and field‐composition comparisons help delimit this result without establishing absence of genetic effects. Top‐ranked LD‐clumped regions remain exploratory candidates requiring independent validation. Larger populations, independent field evaluations, and consistent plot‐level phenotyping are needed for stronger locus‐level conclusions.

## Funding

This work was supported by U.S. Department of Agriculture, Agricultural Research Service, No. 8042‐21220‐258‐000‐D, 8042‐21000‐303‐000‐D.

## Conflicts of Interest

The authors declare no conflicts of interest.

## Supporting information


**Data S1:** Leaf‐blight plot records, accession phenotypes and inclusion definitions; score/LRT/Wald profiles; transformation diagnostics and the 8566‐marker PC basis; concordance and rank overlap; ACAT–Simes comparisons; exploratory candidate statistics and nearby genes; detectable‐effect calculations; genotype‐equivalence and LD‐clumping summaries; and model, leave‐one‐location‐out and matched‐estimator effect‐direction comparisons. A README and data dictionary define each worksheet and its interpretation.

**Figure S1:** Phenotype coverage and quality‐control summary. (a) Complete incidence–severity plot pairs at the five locations (1339 records in total). (b) Number of locations represented for each of the 104 phenotyped identifiers; primary genomic inference used 100 genotype‐matched accessions represented in at least four locations. The vertical dashed line in (b) marks the four‐location inclusion threshold. (c) Distribution of the number of complete plot records per accession. (d) Recorded plot‐level incidence and severity percentages; overlapping points can represent multiple records. Numeric zero denotes recorded absence of disease, not a missing value.
**Figure S2:** Genomic principal‐component structure of the primary 100‐accession population. (a) PC1 versus PC2. (b) PC1 versus PC3. Principal components were obtained from the variance‐standardized, LD‐pruned structure‐marker matrix. Each point is an accession. Colors identify the *N* and *S* accession‐prefix groups and entries labeled as checks; they are identifiers, not independently inferred genetic clusters. Both panels use the same color key.
**Figure S3:** Quantile–quantile profiles for official GEMMA 0.98.5 LOCO score tests. Panels show (a) incidence, (b) standard severity, (c) ACAT and (d) Simes. Observed −log_10_(*p*) values are plotted against the uniform‐null expectation for 567,758 marker rows. Diagonal lines denote equality, and insets report the median‐based genomic inflation factor *λ*_GC_. Linked and genotype‐equivalent markers remain represented in these plots. Near‐null medians alone do not establish absence of confounding or correct extreme‐tail calibration.
**Figure S4:** Sensitivity to the association statistic. (a) Numbers of marker rows passing Benjamini–Hochberg *q* < 0.10 under score, likelihood‐ratio (LRT) and Wald tests for incidence (INC), severity (SEV), ACAT and Simes. Score and LRT counts are zero; the visible non‐zero bars are Wald results. (b) Genome‐wide Spearman correlations between the corresponding −log_10_(*p*) profiles. Large rank correlations can coexist with marked disagreement in the extreme tail. Score results supply the primary summaries, LRT results the sensitivity comparison, and Wald results the diagnostic comparison. The test comparison does not by itself establish which test has the correct false‐positive rate.
**Figure S5:** Population‐structure and sample‐inclusion sensitivity of score‐test profiles. (a) Spearman correlations of −log_10_(*p*) with the primary kinship‐only, 100‐accession analysis. Comparators add three genomic principal components (*K* + PC3) or retain all 102 genotype‐matched accessions. Lines connect the four methods for visual comparison and do not represent a continuous variable. (b) The strongest association, −log_10_(minimum *p*), in each comparator. Colors consistently identify the two comparisons within each panel. INC, incidence; SEV, standard severity. These are sensitivity comparisons, not independent replication datasets.
**Figure S6:** Leave‐one‐location‐out concordance of genome‐wide score‐test profiles. Columns identify the omitted field location; rows show incidence (INC), standard severity (SEV), ACAT and Simes. Each cell gives the Spearman correlation with the corresponding all‐location profile across marker rows, rounded to two decimal places. Color encodes the same correlation on a common 0–1 scale. Field‐adjusted accession values and association profiles were reconstructed after each omission. The datasets overlap substantially, so the comparison measures sensitivity to field composition rather than independent replication.
**Figure S7:** Regional score‐based ACAT profiles for four exploratory candidate entries. Panels show (a) S05_34711017 on chromosome 5, (b) S09_24644668 on chromosome 9, (c) S09_53491107 on chromosome 9 and (d) S06_35920649 on chromosome 6, each within ±1 Mb of its index marker. The ordinate is −log_10_(*p*); the abscissa is physical position in base pairs, with the displayed 10^7^ multiplier. The vertical dashed line marks the index marker. Point color and each color bar give squared dosage correlation *r* ^2^ with that marker in the primary genotypes. These are top‐ranked exploratory profiles, not FDR‐significant loci or fine‐mapping results.
**Figure S8:** Illustrative detectable‐effect requirements in the primary *n* = 100 population. Panels show (a) 80% and (b) 90% power under a two‐sided noncentral‐*t* approximation. Blue curves use *α* = 0.05/567,758 marker rows; orange curves use *α* = 0.05/455,783 chromosome‐specific genotype classes; green curves use a descriptive reference *p* = 10^−5^. The two Bonferroni curves nearly coincide. Effects are expressed as residual standard deviations per allele, *β*_res_ = *β*/*σ*_e_, under the assumed genotype variance 2*f*(1 − *f*), where *f* is minor‐allele frequency. The approximation uses *n* − 2 degrees of freedom and independent observations. It does not include kinship, inbreeding‐related departures from the assumed variance, or uncertainty in field‐adjusted phenotypes. It is not empirical power for the fitted LOCO model or a measure of observed variance explained. Results for *n* = 102 are also included in Data S1 (Power).

## Data Availability

The analysis code, including the auxiliary diagnostic routines, is available at https://github.com/EJSAHN/sorghum‐leaf‐blight‐meta‐gwas and archived in Zenodo (version 2.0.2; DOI: https://doi.org/10.5281/zenodo.22446088). Field records derive from the Senegal evaluations reported by Prom et al. (2023) and the Niger evaluations reported by Prom et al. (2026). The public genotype resource is available from the Texas Data Repository (DOI: https://doi.org/10.18738/T8/RGPPGA). Data S1 contains the leaf‐blight plot records, accession estimates, sample‐inclusion definitions and processed result summaries used for the figures and tables. Raw genotype data are not included in the software archive.
