# Supplementary material for: Multi‐Environment Genomic Analysis Reveals Limited Concordance Between Sorghum Leaf Blight Incidence and Severity
Source: Plant Environ Interact. 2026 Nov 2;7(6):e70214. doi: 10.1002/pei3.70214 (PMC13728317; doi:10.1002/pei3.70214)
Supplement: Supplementary file 1 [file PEI3-7-e70214-s001.docx]

**Supplementary Figure S1**

**Figure S1. Phenotype coverage and quality-control summary.** (a) Complete incidence–severity plot pairs at the five locations (1,339 records in total). (b) Number of locations represented for each of the 104 phenotyped identifiers; primary genomic inference used 100 genotype-matched accessions represented in at least four locations. The vertical dashed line in (b) marks the four-location inclusion threshold. (c) Distribution of the number of complete plot records per accession. (d) Recorded plot-level incidence and severity percentages; overlapping points can represent multiple records. Numeric zero denotes recorded absence of disease, not a missing value.

**Supplementary Figure S2**

**Figure S2. Genomic principal-component structure of the primary 100-accession population.** (a) PC1 versus PC2. (b) PC1 versus PC3. Principal components were obtained from the variance-standardised, LD-pruned structure-marker matrix. Each point is an accession. Colours identify the *N* and *S* accession-prefix groups and entries labelled as checks; they are identifiers, not independently inferred genetic clusters. Both panels use the same colour key.

**Supplementary Figure S3**

**Figure S3. Quantile–quantile profiles for official GEMMA 0.98.5 LOCO score tests.** Panels show (a) incidence, (b) standard severity, (c) ACAT and (d) Simes. Observed −log_10_(*p*) values are plotted against the uniform-null expectation for 567,758 marker rows. Diagonal lines denote equality, and insets report the median-based genomic inflation factor *λ*_GC_. Linked and genotype-equivalent markers remain represented in these plots. Near-null medians alone do not establish absence of confounding or correct extreme-tail calibration.

**Supplementary Figure S4**

**Figure S4. Sensitivity to the association statistic.** (a) Numbers of marker rows passing Benjamini–Hochberg *q* < 0.10 under score, likelihood-ratio (LRT) and Wald tests for incidence (INC), severity (SEV), ACAT and Simes. Score and LRT counts are zero; the visible non-zero bars are Wald results. (b) Genome-wide Spearman correlations between the corresponding −log_10_(*p*) profiles. Large rank correlations can coexist with marked disagreement in the extreme tail. Score results supply the primary summaries, LRT results the sensitivity comparison, and Wald results the diagnostic comparison. The test comparison does not by itself establish which test has the correct false-positive rate.

**Supplementary Figure S5**

**Figure S5. Population-structure and sample-inclusion sensitivity of score-test profiles.** (a) Spearman correlations of −log_10_(*p*) with the primary kinship-only, 100-accession analysis. Comparators add three genomic principal components (*K*+PC3) or retain all 102 genotype-matched accessions. Lines connect the four methods for visual comparison and do not represent a continuous variable. (b) The strongest association, −log_10_(minimum *p*), in each comparator. Colours consistently identify the two comparisons within each panel. INC, incidence; SEV, standard severity. These are sensitivity comparisons, not independent replication datasets.

**Supplementary Figure S6**

**Figure S6. Leave-one-location-out concordance of genome-wide score-test profiles.** Columns identify the omitted field location; rows show incidence (INC), standard severity (SEV), ACAT and Simes. Each cell gives the Spearman correlation with the corresponding all-location profile across marker rows, rounded to two decimal places. Colour encodes the same correlation on a common 0–1 scale. Field-adjusted accession values and association profiles were reconstructed after each omission. The datasets overlap substantially, so the comparison measures sensitivity to field composition rather than independent replication.

**Supplementary Figure S7**

**Figure S7. Regional score-based ACAT profiles for four exploratory candidate entries.** Panels show (a) S05_34711017 on chromosome 5, (b) S09_24644668 on chromosome 9, (c) S09_53491107 on chromosome 9 and (d) S06_35920649 on chromosome 6, each within ±1 Mb of its index marker. The ordinate is −log_10_(*p*); the abscissa is physical position in base pairs, with the displayed 10^7^ multiplier. The vertical dashed line marks the index marker. Point colour and each colour bar give squared dosage correlation *r*^2^ with that marker in the primary genotypes. These are top-ranked exploratory profiles, not FDR-significant loci or fine-mapping results.

**Supplementary Figure S8**

**Figure S8. Illustrative detectable-effect requirements in the primary *n* = 100 population.** Panels show (a) 80% and (b) 90% power under a two-sided noncentral-*t* approximation. Blue curves use *α* = 0.05/567,758 marker rows; orange curves use *α* = 0.05/455,783 chromosome-specific genotype classes; green curves use a descriptive reference *p* = 10^−5^. The two Bonferroni curves nearly coincide. Effects are expressed as residual standard deviations per allele, *β*_res_ = *β*/*σ*_e_, under the assumed genotype variance 2*f*(1 − *f*), where *f* is minor-allele frequency. The approximation uses *n* − 2 degrees of freedom and independent observations. It does not include kinship, inbreeding-related departures from the assumed variance, or uncertainty in field-adjusted phenotypes. It is not empirical power for the fitted LOCO model or a measure of observed variance explained. Results for *n* = 102 are also included in Supplementary Data S1 (Power).
